# Pathophysiology of Hypoperfusion of the Precuneus in Early Alzheimer's Disease

**DOI:** 10.1111/bpa.12331

**Published:** 2015-11-09

**Authors:** J. Scott Miners, Jennifer C. Palmer, Seth Love

**Affiliations:** ^1^ Dementia Research Group School of Clinical Sciences, Institute of Clinical Neurosciences, University of Bristol Bristol UK

**Keywords:** Alzheimer's disease, amyloid‐β, blood flow, endothelin‐1, oxygenation, precuneus

## Abstract

The earliest decline in cerebral perfusion in Alzheimer's disease (AD) is in the medial parietal cortex (precuneus). We have analyzed precuneus in post‐mortem tissue from 70 AD and 37 control brains to explore the pathophysiology of the hypoperfusion: the contribution of arteriolosclerotic small vessel disease (SVD) and cerebral amyloid angiopathy (CAA), and of the vasoconstrictors endothelin‐1 (EDN1) and angiotensin II (Ang II), and the association with Aβ. The myelin‐associated glycoprotein:proteolipid protein‐1 ratio (MAG:PLP1) was used as an indicator of oxygenation of the precuneus prior to death. MAG:PLP1 was reduced ∼50% in early AD (Braak stage III–IV). Although MAG:PLP1 remained low in advanced AD (stage V–VI), the reduction was less pronounced, possibly reflecting falling oxygen demand. Reduction in cortical MAG:PLP1 correlated with elevation in vascular endothelial growth factor (VEGF), another marker of hypoperfusion. Cortical MAG:PLP1 declined nonsignificantly with increasing SVD and CAA, but significantly with the concentration of EDN1, which was elevated approximately 75% in AD. In contrast, with reduction in cortical MAG:PLP1, Ang II level and angiotensin‐converting enzyme (ACE) activity declined, showing a normal physiological response to hypoperfusion. MAG:PLP1 was reduced in the parietal white matter (WM) in AD but here the decline correlated positively (ie, physiologically) with WM EDN1. However, the decline of MAG:PLP1 in the WM was associated with increasing cortical EDN1 and perhaps reflected vasoconstriction of perforating arterioles, which traverse the cortex to perfuse the WM. EDN1 in the cortex correlated highly significantly with both soluble and insoluble Aβ42, shown previously to upregulate neuronal endothelin‐converting enzyme‐2 (ECE2), but not with Aβ40. Our findings demonstrate reduced oxygenation of the precuneus in early AD and suggest that elevated EDN1, resulting from Aβ42‐mediated upregulation of ECE2, is a contributor.

## INTRODUCTION

Blood flow and glucose utilization decline in the precuneus at a very early stage of Alzheimer's disease (AD) 2, 6, 7, 13, 28, 30, 53. Reduction in cerebral blood flow precedes the development of dementia in AD 52 and occurs well before any behavioral or pathological abnormalities in animal models of the disease 24, 39. We still have only a limited understanding of the pathogenesis.

Cerebrovascular abnormalities are common in AD 26, up to 60% of patients having ischaemic WM damage 9, 10, 17, 26 and over 90% having cerebral amyloid angiopathy (CAA) 16, 18, 29. Brain ischaemia is the defining pathological process in vascular dementia but there is evidence that ischaemia has the potential to contribute to the development of AD pathology. Ischaemia in animal models, or its simulation by deprivation of oxygen and glucose *in vitro*, is associated with increased production of Aβ (reviewed in 12). Transient global cerebral ischaemia due to cardiac arrest in man was shown to cause a significant rise in serum Aβ42, lasting several days 61. Serum Aβ42 also rose in people who had sustained diffuse traumatic brain injury 37, in which there is invariably brain swelling and reduced perfusion; the rise in serum Aβ42 was accompanied by a decline in Aβ42 in the cerebrospinal fluid, arguing against nonspecific “leakage” of Aβ42 from damaged brain tissue. There is also strong evidence from observational and experimental studies that CAA and arteriolosclerotic small vessel disease (SVD) impede the clearance of interstitial solutes (including Aβ) from the brain 20, 21, 22, 59, 60. Hughes *et al* 23 found that arterial stiffness in elderly nondemented people correlated with the amount of cerebral Aβ, as demonstrated by Aβ‐positron emission tomography. Kester *et al* 27 showed that the level of Aβ42 in the CSF in nondemented elderly people was lower in those with ischaemic WM abnormalities on magnetic resonance imaging (reduction in Aβ42 in the CSF being associated with increased AD pathology).

Conversely, there is strong evidence that Aβ peptides cause reduction in cerebral blood flow—not only through the development of CAA but also by inducing vasoconstriction. In animal studies, Aβ peptides reduced cerebral blood flow, interfered with cerebral autoregulation and impeded functional hyperaemia 39, 40, 41. We previously demonstrated an increase in the level of the vasoconstrictor peptide endothelin‐1 (EDN1) in the cerebral cortex in AD 46. We showed that Aβ40 upregulated endothelin‐converting enzyme‐1 (ECE1)‐mediated production of EDN1 by cerebrovascular endothelial cells 48, and that Aβ42 upregulated endothelin‐converting enzyme‐2 (ECE2)‐mediated production of EDN1 by neurons 45. Another vasoconstrictor with the potential to exacerbate cerebral hypoperfusion in AD is angiotensin II (Ang II), cleaved from angiotensin I by the action of angiotensin‐converting enzyme (ACE), the level of which was found to be elevated in the frontal cortex in AD 31, 35. ECE1, ECE2 and ACE are all capable of cleaving Aβ, and their upregulation in AD is probably a response to the accumulation of Aβ substrate 33.

We recently developed a novel method to quantify ischaemic damage in post‐mortem brain tissue, by comparison of the levels of two myelin proteins: myelin‐associated glycoprotein (MAG), which is highly susceptible to reduced tissue oxygenation, and proteolipid protein‐1 (PLP‐1), which is relatively resistant 3, 4, 56. In frontal cortex from patients with AD, we found the MAG:PLP1 ratio to be significantly reduced, indicating a pathological reduction in perfusion (ie, reduction exceeding the decline in metabolic demand) 56. Although MAG:PLP1 tended to be lower in cortex from patients with severe SVD or CAA, the only significant negative correlation was with the concentration of EDN1.

In this study, we have used similar methods to identify contributors to cerebral hypoperfusion in very early AD, by examining the precuneus, a region that is amongst the first affected by hypoperfusion, and by analyzing the findings in relation to the progression of AD, as indicated by the Braak tangle stage 8. We have found evidence of pathological hypoperfusion of the precuneus at an early stage of AD, associated with elevation of EDN1 and correlating closely with the level of Aβ42. Our findings suggest a key role for Aβ42‐mediated upregulation of ECE2 in the reduction of cerebral perfusion and oxygenation in early AD.

## MATERIALS AND METHODS

### Case selection

From the South West Dementia Brain Bank, University of Bristol, we obtained tissue from 70 cases of AD (ages 57–99 years, mean 79.8 years, SD 8.3 years) with post‐mortem delays of 4–72 h (mean 31.4 h, SD 19.3 h). All of the brains had been subjected to detailed neuropathological assessment, and according to the NIA‐AA guidelines 38 AD pathology was a sufficient explanation for the dementia in these cases. We also obtained tissue from 37 control brains that had also been extensively assessed neuropathologically, from people who had no history of dementia, few or absent neuritic plaques, a Braak tangle stage of III or less and no other neuropathological abnormalities apart from scattered diffuse plaques in most cases. Their ages ranged from 58 to 94 y (mean 79.8 years, SD 8.7 years) and the post‐mortem delays from 3 to 67 h (mean 35.0 h, SD 15.4 h). The cohorts overlapped those in a previous study of deep parietal WM 4. For analysis of the effect of stage of AD on MAG:PLP1 in the precuneus, the AD and control cohorts were pooled and cases were subdivided according to Braak tangle stage (0–II, III–IV and V–VI) irrespective of the presence or absence of a history of dementia. The demographic data, neuropathological findings, and MRC identifier numbers in this cohort are summarized in Supporting Information Tables S1 and S2. The study had local research ethics committee approval.

### Brain tissue

The brains had been obtained within 72 h of death. The right cerebral cortex had been fixed in 10% formalin for three weeks before the tissue was processed and paraffin blocks were taken for pathological assessment. SVD had been scored as previously described 4, on a four‐point semiquantitative scale according to the extent of thickening of the arteriolar walls and associated narrowing of the vessel lumina: 0 = normal vessel wall thickness, 1 = slightly increased thickness, 2 = moderately increased thickness and 3 = markedly increased thickness such that for many arterioles the diameter of the lumen was <50% of the outer diameter of the blood vessel. CAA for all cases had also been previously graded semiquantitatively on a four‐point scale by a method adapted from that of Olichney *et al* 11, 42, ranging from “0” for vessels devoid of amyloid to “3” for extensive deposition. The left cerebral hemisphere had been sliced and frozen at −80°C until used for biochemical assessment. Tissue was dissected from the medial parietal cortex (Brodmann area 7) and separate samples were dissected from the underlying parietal WM. Biochemical analyses were performed on 200 mg samples of the dissected tissue that were homogenized in 1% sodium dodecyl sulfate lysis buffer in a Precellys homogenizer (Stretton Scientific, Derbyshire, UK) and then aliquoted and stored at −80°C until required. All measurements were made in duplicate and the mean determined.

### Measurement of MAG by direct ELISA

MAG level was measured by direct ELISA, as previously described 3, 4, 36, 56. Brain tissue homogenates in PBS (1:10) and PBS blanks were left for 2 h at room temperature with constant shaking in 96‐well microplates. The plate was washed and blocked in 1% bovine serum albumin/PBS for 2 h at room temperature and, after further washes, incubated for 2 h at room temperature with mouse monoclonal anti‐MAG antibody (Abcam, Cambridge, UK) diluted 1:1000 in PBS. The plate was washed, incubated with biotin‐conjugated anti‐mouse secondary antibody (Vector Labs, Peterborough, UK) diluted 1:500 in PBS for 20 minutes, followed by another wash step and incubation with streptavidin‐horseradish peroxidase (R&D systems, Oxford, UK), 1:500 in PBS, for 20 minutes in the dark. The plate was washed and incubated for 10 minutes in the dark with 100 μL/well of chromogenic substrate (TMBS substrate, R&D systems, Oxford, UK). The absorbance was read at 450 nM in a FLUOstar Optima plate reader(BMG Labtech, Aylesbury, UK) after the addition of 50 μL of 2 N sulfuric acid. MAG level was determined for each case by interpolation against a standard curve generated by serial dilution (400–6.25 ng/mL) of recombinant human MAG (Abnova, Taipei City, Taiwan). We previously demonstrated that MAG is stable under conditions of simulated post‐mortem delay for up to 72 h at 4°C or room temperature 4.

### Measurement of PLP1 by sandwich ELISA

PLP1 level was measured in brain tissue homogenates by use of a commercially available sandwich ELISA (cat no SEA417Hu, USCN, Wuhan, China) as described previously 56. Brain tissue homogenates were diluted 1:10 in PBS. Absolute PLP1 level was interpolated from a standard curve generated by serial dilution of recombinant human PLP1 (10–0.156 ng/mL). We previously demonstrated that PLP1 is stable under conditions of simulated post‐mortem delay for up to 72 h at 4°C or room temperature 4.

### Measurement of EDN1 by sandwich ELISA

EDN1 level was measured in brain tissue homogenates, diluted to 1 mg/mL, by use of the QuantiGlo Chemiluminescent ELISA kit for human EDN1 (R&D Systems, Oxford, UK) as previously described 3, 36, 46, 48, 56. Relative luminescence was measured using a in a FLUOstar Optima plate reader. Absolute EDN1 level was interpolated from a standard curve generated by assaying serial dilutions of recombinant human EDN1 (250–0.340 pg/mL). We previously demonstrated that EDN1 is stable under conditions of simulated post‐mortem delay for up to 72 h at 4°C or room temperature 48.

### Measurement of angiotensin II by sandwich ELISA

Human angiotensin II ELISA kit (Abcam, Cambridge, UK) was used to measure Ang II level in brain tissue homogenates according to the manufacturer's guidelines. Ang II was measured in 50 μL aliquots of brain tissue homogenate, adjusted for total protein, and the concentration determined by interpolation against a standard curve generated by serial dilution of recombinant human Ang II (1000–63 pg/mL). Absorbance was read at 450 nM in a FLUOstar Optima plate reader.

### Measurement of VEGF by sandwich ELISA

Vascular endothelial growth factor (VEGF) level was measured in 10 μL of brain tissue homogenate plus 90 μL PBS by use of Human VEGF Quantikine ELISA kit (R&D Systems, Oxford, UK), as previously described 36, 56. The ELISA used a monoclonal mouse VEGF antibody as a capture antibody and a polyclonal biotinylated VEGF detection antibody. Absorbance was measured at 450 nm in a FLUOstar Optima plate reader after the addition of 50 μL of 2N sulfuric acid. Absolute VEGF level was interpolated from a serial dilution of recombinant human VEGF (2000–31.25 pg/mL).

### Measurement of A**β**40 and A**β**42 sandwich ELISA

We prepared soluble and insoluble (guanidine‐extractable) fractions of the homogenates for Aβ measurement as reported in previous studies 1, 5, 32, 55, 56, 57, 58.

For measurement of Aβ40 we used mouse anti‐human Aβ (clone 6E10, raised against amino acids 1–16; Covance, 2 µg/mL) as the capture antibody and mouse anti‐human Aβ40 (11A50‐B10, Covance, 1 µg/mL) as the detection antibody, after biotinylating the Aβ40 antibody by use of Lightning‐Link® Biotinylation Kit (Innova Biosciences, Cambridge, UK). The soluble and insoluble fractions of brain homogenate (diluted 1:3 and 1:49, respectively), and serial dilutions of recombinant human Aβ40 (Sigma Aldrich, Dorset, UK) in PBS containing 1% 1,10 phenanthroline (Sigma Aldrich) to prevent degradation of Aβ 51, were incubated for 2 h at room temperature on a rocking platform. Absorbance was read at 450 nm in a FLUOstar plate reader.

For measurement of Aβ42 we used mouse anti‐human Aβ42 (Covance 12F4, 1 µg/mL) (Cambridge Biosciences, Cambrideg, UK) as the capture antibody and biotinylated anti‐human Aβ (10H3, 0.1 μg/mL) (Thermo Fisher Scientific, Loughborough, UKas the detection antibody. The soluble and insoluble fractions, diluted as above, and serial dilutions of human recombinant Aβ42 (16 000 to 1.024 nM) in PBS containing 1% 1,10 phenanthroline were incubated for 2 h at room temperature on a rocking platform. Aβ42 concentration in brain tissue was determined by interpolation against a standard curve generated by serial dilution of recombinant human Aβ42 (Sigma Aldrich, Dorset, UK). Each sample was assayed in duplicate. The Aβ40 ELISA did not show any cross‐reactivity with recombinant Aβ42, nor did the Aβ42 ELISA with recombinant Aβ40.

### Measurement of ACE‐1 activity

ACE‐1 activity in brain homogenates was measured by immunocapture‐based fluorogenic assay, as previously described 1, 3, 25, 36, 56. ACE/CD143 antibody (5 ng/mL in PBS) (R&D systems, Oxford, UK) was coated on a black Fluoronunc plate overnight then blocked in 1% BSA/PBS for 2 h at room temperature before addition of 10 μL tissue homogenate plus 90 μL PBS, or serial dilutions of recombinant human ACE, and incubation for 2 h at room temperature with constant shaking. Fluorogenic peptide substrate (ES005, R&D systems, Oxford, UK) diluted in activity assay buffer (100 mM Tris‐HCl pH 7.5, 50 mM NaCl, 10 µM ZnCl_2_) was added and the plate incubated for 3 h at 37°C in the dark. Fluorescence was measured at 405 nm with excitation at 320 nm. To determine ACE‐specific enzyme activity we subtracted the fluorescent signal from that after inhibition by captopril (Enzo Life Sciences, Exeter, UK). ACE activity was interpolated from a standard curve produced by serial dilution of recombinant human ACE (2500–39 pg/mL) (R&D systems, Oxford, UK).

### Statistical analysis

Unpaired two‐tailed *t* tests or ANOVA with Dunnett's *post hoc* analysis was used for comparisons between groups, and Pearson's or Spearman's test to assess linear or rank order correlation, as appropriate, with the help of SPSS version 16 (SPSS, Chicago) and GraphPad Prism version 6 (GraphPad Software, La Jolla, CA). *P*‐values < 0.05 were considered statistically significant.

## RESULTS

### Oxygenation of the precuneus is decreased in early Alzheimer's disease

**Figure 1:**
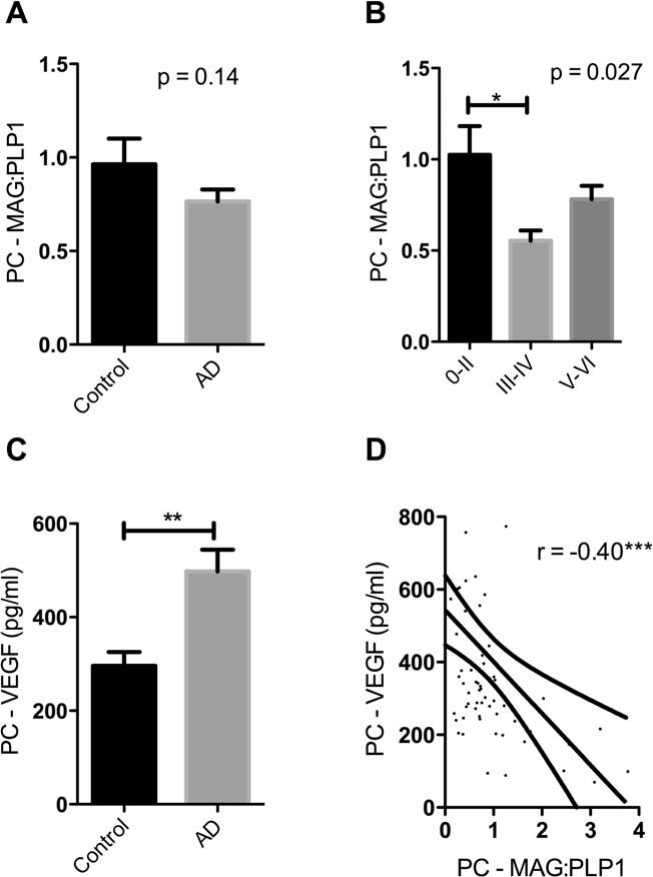
Oxygenation of the precuneus was reduced in early Alzheimer's disease. **A**. Bar chart showing a reduction in the ratio of myelin glycoprotein (MAG) to proteolipid‐1 protein (PLP‐1) (MAG:PLP1) in the precuneus in AD. The bars indicate the mean and SEM. **B**. Bar chart showing marked variation in MAG:PLP1 (*P* = 0.027) with disease stage. For this analysis, control and AD cases were combined and grouped according to Braak tangle stage (0–II, III–IV and V–VI). *Post hoc* analysis revealed that MAG:PLP1 was significantly reduced in early AD (Braak stage III–IV) compared to controls (*P* = 0.027). **C**. Bar chart showing elevated VEGF, an independent marker of cerebral perfusion, in AD. **D**. Scatterplot showing the highly significant negative correlation between MAG:PLP1 and VEGF concentration in the precuneus (*r* = −0.40, *P* = 0.0007). The best‐fit linear regression line and 95% confidence interval are superimposed. **P < 0.001, ***P < 0.0001.

MAG:PLP1 in the precuneus was lower in the AD than the control cohort but not significantly so (*P* = 0.14) (Figure 1A). However, MAG:PLP1 varied significantly according to Braak tangle stage (Figure 1B) (*P* = 0.027). The highest values were in the Braak stage 0–II group and the lowest (by about 50%) in the Braak stage III–IV group. *Post hoc* analysis revealed that the difference between the Braak stage 0–II and III–IV groups was statistically significant (*P* = 0.021, Dunnett's test) whereas the difference between the Braak stage 0–II and V–VI groups did not reach significance (*P* = 0.092).

Our previous studies showed that VEGF level increases in hypoperfused brain tissue, is elevated in AD frontal cortex and correlates inversely with MAG:PLP1 3, 4, 56. In the precuneus too, VEGF was elevated in AD (Figure 1C), rose with Braak tangle stage (Supporting Information Figure S1A) and correlated inversely with MAG:PLP1 (*r* = −0.40 *P* < 0.001) (Figure 1D). VEGF level correlated with insoluble Aβ42 (*r* = 0.41 *P* < 0.01) (Supporting Information Figure S1B) but not insoluble Aβ40 (Supporting Information Figure S1C).

### Oxygenation of precuneus in AD is not significantly affected by small vessel disease or cerebral amyloid angiopathy

**Figure 2:**
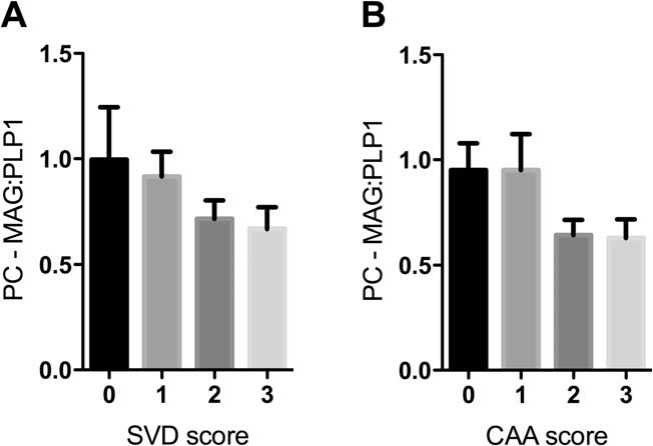
MAG:PLP1 tended to decline in relation to severity of SVD and CAA in the precuneus in AD. Bar charts show lower MAG:PLP1 in precuneus from AD patients with higher severity scores for SVD (**A**) and CAA (**B**), although the differences were not statistically significant.

We next examined whether the changes in MAG:PLP1 in the precuneus in AD were attributable to SVD or CAA. MAG:PLP1 declined as SVD (Figure 2A) and CAA (Figure 2B) increased in severity. However, the relationship between MAG:PLP1 and the SVD or CAA severity score was not statistically significant.

### Endothelin‐1 is increased in the precuneus in AD in association with reduced tissue oxygenation

**Figure 3:**
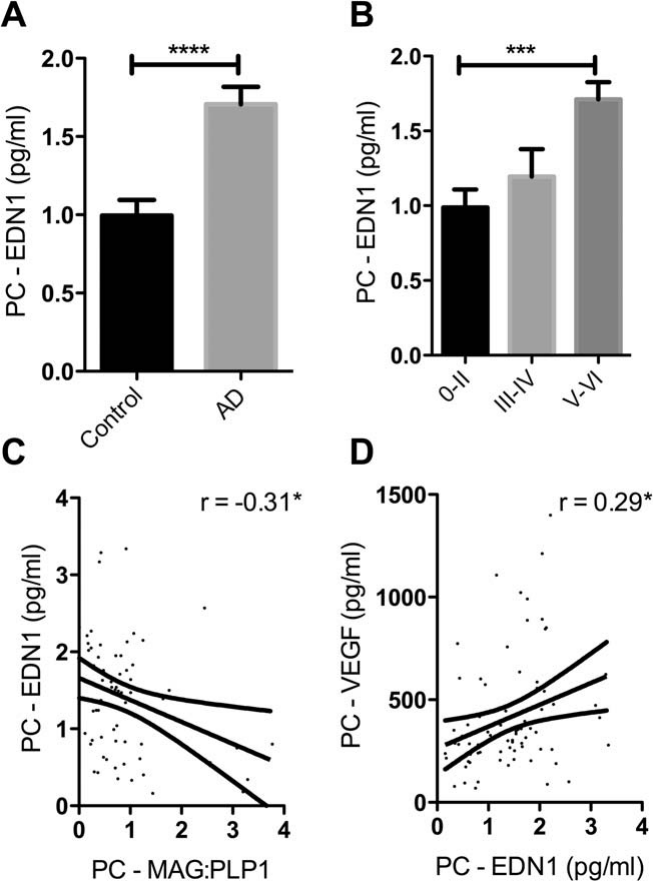
Reduced oxygenation of the precuneus in AD was associated with elevated EDN1. **A**. Bar chart showing significantly increased EDN1 in AD within the precuneus. **B**. Bar chart showing increased EDN1 levels in relation to disease severity when control and AD cases were subdivided according to Braak tangle stage (0–II, III–IV and V–VI) irrespective of the presence or absence of a history of dementia. Scatterplots showing the inverse correlation between EDN1 concentration and MAG:PLP1 ratio (*r* = −0.31) (**C**) and the positive correlation between EDN1 and VEGF (*r* = 0.29) (**D**). *P < 0.05, ***P < 0.001, ****P < 0.0001.

We previously reported that the concentration of EDN1 is increased in AD in the temporal 46 and frontal cortex 56. EDN1 was also elevated in the precuneus in AD compared to age‐matched controls (*P* < 0.0001) (Figure 3A) and rose significantly with Braak tangle stage (*P* = 0.0003). The level correlated negatively with MAG:PLP1 (*r* = −0.31, *P* < 0.05) (Figure 3C) and positively with VEGF (*r* = 0.29, *P* < 0.05) (Figure 3D).

### ACE‐1 activity and Ang II level declined with reduced oxygenation of the precuneus

**Figure 4:**
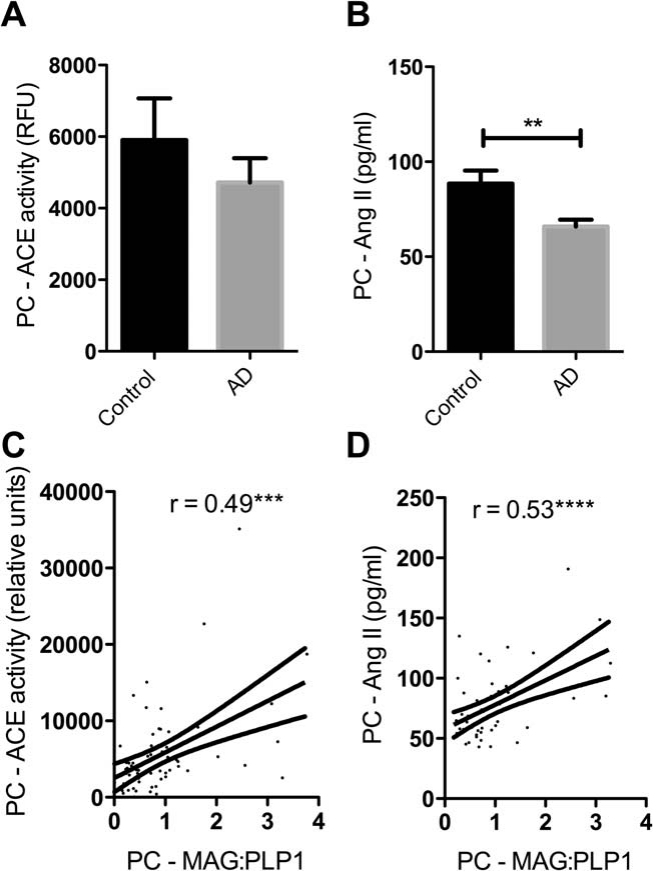
ACE activity and angiotensin II (Ang II) levels declined in association with reduced oxygenation of the PC. **A**. Bar charts showing a nonsignificant reduction in ACE activity and (**B**) significantly reduced Ang II in the precuneus in AD. Scatterplots show that (**C**) ACE activity (*r* = 0.494) and (**D**) Ang II (*r* = 0.53) correlated positively with MAG:PLP1. ***P<0.001, ****P<0.0001.

In previous post‐mortem studies, ACE‐1 activity was elevated in midfrontal cortex in AD 31, 34, 35. In contrast, both ACE activity (Figure 4A) and the level of its cleavage product Ang II (Figure 4B) were reduced in the precuneus in AD, although only the difference in Ang II concentration was significant (*P* = 0.004). ACE activity varied with Braak tangle stage although not significantly, in a pattern resembling the variation in MAG:PLP1 (Supporting Information Figure S1D). ACE and Ang II level correlated closely with cortical MAG:PLP1 (Figures 4C and D), as would be expected physiologically: Ang II production declining under conditions of reduced oxygenation, to minimize the risk of ischaemic damage.

### White matter oxygenation and EDN1

**Figure 5:**
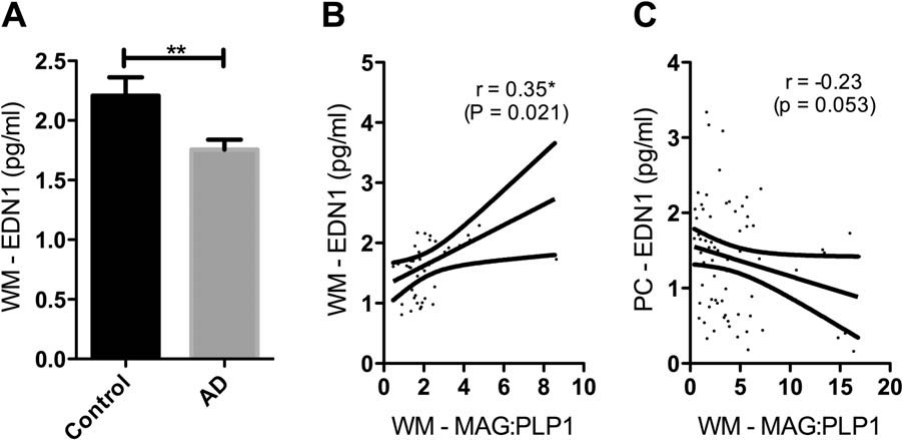
EDN1 level was reduced in the WM in AD, the reduction correlating with the fall in oxygenation. **A**. Bar chart showing reduced EDN1 in the parietal WM (*P* = 0.006). Scatterplots show the positive correlation between WM MAG:PLP and WM EDN1 (*r* = 0.35, *P* = 0.021) (**C**) and the negative correlation between WM MAG:PLP and PC EDN1 in (*r* = −0.23, *P* = 0.053) (**D**). *P < 0.05, **P < 0.01.

We had previously measured MAG:PLP1 in the parietal WM 3 and now measured EDN1 concentration in the same homogenates. In contrast to the elevated EDN1 in the cortex in AD, EDN1 concentration in the WM was significantly reduced (Figure 5A), the concentration correlating with MAG:PLP1 in the WM (Figure 5B) in keeping with a physiological response to inadequate perfusion.

In contrast, WM MAG:PLP1 correlated negatively with EDN1 in the cortex (*r* = −0.23, *P* = 0.053), suggesting that the ratio was affected by EDN1‐mediated vasoconstriction of the perforating arterioles that transverse the cortex to perfuse the WM.

### EDN1 level was related to A**β**42 accumulation in the precuneus

**Figure 6:**
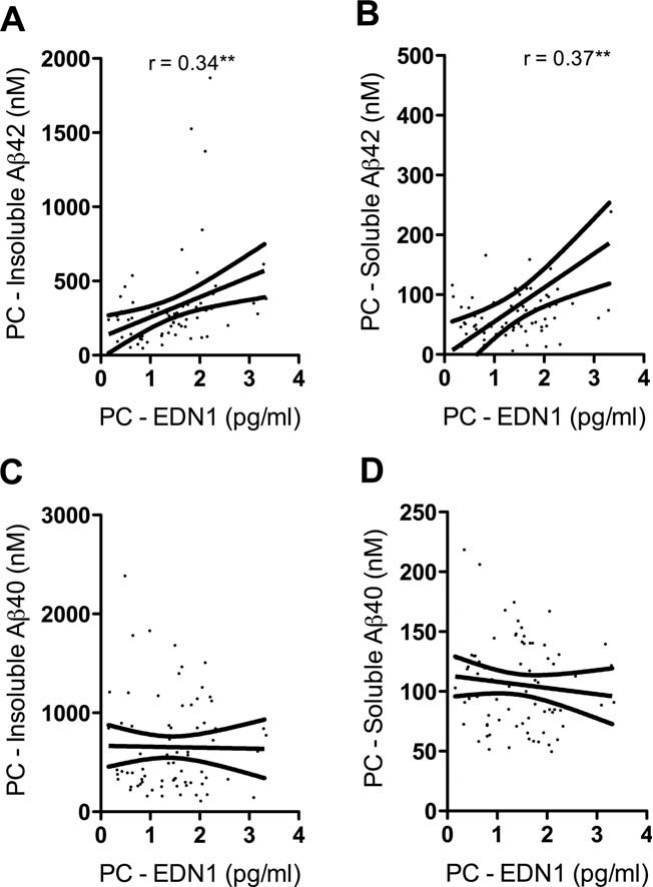
EDN1 level correlated with Aβ42 in the precuneus (PC). PC EDN1 correlated with the level of insoluble (*r* = 0.34) (**A**) and soluble Aβ42 (*r* = 0.37) (**B**) but not the level of insoluble (**C**) or soluble (**D**) Aβ40. **P < 0.01.

EDN1 level within the precuneus correlated positively with insoluble Aβ42 (*r* = 0.34, *P* < 0.01) (Figure 6A) and soluble Aβ42 (*r* = 0.37, *P* < 0.01) (Figure 6B) but not with soluble or insoluble Aβ40 (Figure 6C, D). EDN1 level in the underlying WM did not correlate with insoluble or soluble Aβ42 or Aβ40 in either region.

## DISCUSSION

Our previous studies on post‐mortem samples of frontal lobe revealed pathological hypoperfusion of midfrontal cerebral cortex in AD 3, 4, 56, associated with an increase in cortical EDN1. The present findings indicate that these changes are not simply late‐stage manifestations of AD but are demonstrable even in early AD (ie, in Braak stage III–IV disease) in the precuneus, a region that is amongst the first affected by hypoperfusion 2, 6, 7, 13, 28, 30, 53. Indeed, the MAG:PLP1 ratio, an indicator of the adequacy of ante‐mortem tissue oxygenation, was lower in early than late AD (Braak stage V–VI) when the reduction was less pronounced, possibly reflecting falling oxygen demand, for example, as a result of reduced synaptic activity. As in the frontal cortex, the reduction in MAG:PLP1 in the precuneus correlated with the concentration of EDN1 and the level of Aβ42, likely to be one of the drivers of EDN1 production within the brain. Finally, our measurements on the parietal WM suggest that elevated cortical EDN1 in AD may reduce subcortical WM perfusion as well.

In previous studies, we explored the potential contribution of several structural vascular abnormalities to reduced oxygenation of the cerebral cortex and WM in AD 3, 4, 56. SVD and CAA probably contribute to the reduction in blood flow in some patients. In the present study MAG:PLP1 tended to decline with SVD and CAA scores but not significantly. The variability in severity of SVD and CAA across the cohort as a whole may have obscured the contribution that these structural diseases of small vessels make to hypoperfusion of cerebral cortex and WM in a small proportion of cases. The present study did not examine microvessel density, another potential influence on cerebral perfusion (eg, in dementia with Lewy bodies (DLB) 36). However, we did not previously find a significant decline in microvessel density in the cerebral cortex in AD 56.

Of the various potential contributors to reduced oxygenation of the precuneus in AD, the strongest candidate in the present study was EDN1, which doubled in concentration in early AD, and correlated negatively with MAG:PLP1 and positively with the concentration of VEGF. In contrast, the activity of ACE, which catalyses the production of another vasoconstrictor angiotensin‐II, correlated positively with MAG:PLP1, in keeping with a protective vasodilatory response to reduced oxygenation. These findings extend our previous observations on factors influencing oxygenation of the cortex in AD 56. The elevation of EDN1 in the cerebral cortex in AD is not simply a nonspecific consequence of neurodegenerative disease. In DLB, EDN1 level was reduced rather than increased in the occipital cortex 36, a region that is hypoperfused in patients with the disease. Even in the current AD cohort, in which EDN1 was increased in the cortex, it was reduced in the underlying WM, as would be expected physiologically in response to reduced perfusion. Our studies clearly show that the pathophysiology of reduced cerebral perfusion differs not only between dementia subtypes but also between different parts of the brain.

The production of EDN1 is catalyzed by the ECEs. In temporal cortex we found evidence of upregulation of both ECE1 and ECE2 in AD 45, 47. ECE2 is primarily localized to pyramidal cells within the human brain. Our previous studies on human neuroblastoma cells suggest that upregulation of ECE2 in AD and is likely to be a response to the accumulation of Aβ42, as evidenced by the induction of ECE2 on exposure of neuroblastoma cells to human recombinant monomeric or oligomeric Aβ42 but not Aβ40 45. In contrast, in human cerebral endothelial cells Aβ40 upregulated ECE1 and stimulated EDN1 release whereas Aβ42 had no effect 47. In the present study, EDN1 level within the precuneus correlated strongly with the concentration of Aβ42 but not Aβ40.

**Figure 7:**
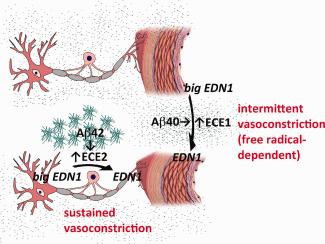
Proposed differences in stimuli and function between ECE2‐ and ECE1‐mediated cerebral vasoconstriction. The present data implicate Aβ42 in the elevated EDN1 level that correlates with a sustained reduction in tissue oxygenation (and therefore of MAG:PLP1) in AD, whereas Aβ40 has been implicated in impairment of transient modulation of arteriolar calibre needed for functional hyperaemia. We reported previously that Aβ42 upregulates ECE2‐mediated production of EDN1 by neurons 45 and that Aβ40 upregulates ECE1‐mediated production of EDN1 by cerebrovascular endothelial cells, a process inhibited by the antioxidant superoxide dismutase 46, 48. Iadecola 24 showed that the transient modulation of arteriolar calibre needed for functional hyperaemia is impaired in mice transgenic for mutant human APP, that this is mediated by Aβ40 and dependent on the production of free radicals. Together these findings suggest that functional hyperaemia is impaired by Aβ40 as a result of a free radical‐mediated increase in ECE1 activity and EDN1 production.

The production of EDN1 by neurons, which is mediated by ECE2 and driven by Aβ42, may cause sustained pathological hypoperfusion of cerebral cortex in AD, particularly in early disease (Figure 7). ECE2 is predominantly localized within the endolysosomal pathway and is responsible for the cleavage of Aβ destined for lysosomal degradation 43. The elevated production of EDN1 may be an unfortunate side effect of over‐activation of this pathway by excessive Aβ42. In contrast, endothelial production of EDN1, mediated by ECE1 and driven by Aβ40, is more likely to contribute to episodic, free radical‐dependent dysfunction of vascular regulation in AD 48 (Figure 7), including abnormalities of autoregulation and functional hyperaemia demonstrated initially in mouse models of cerebral Aβ accumulation 24, 39 and CAA 49, 54, and more recently in patients with AD 14 and probable CAA 50. It should be noted, in addition, that EDN1 is very unlikely to be the sole nonstructural mediator of hypoperfusion of the precuneus in early AD. Other potential contributors include reduced cholinergic vasodilatation, increased production of nitric oxide 19 and, of course reduced synaptic activity/metabolic demand.

Despite progress in our understanding of the pathogenesis of AD there are still only a limited number of available treatment options for patients. There is increasing awareness that, to be effective, most therapies will need to be started at an early stage of disease, before the onset of significant neuropathological changes. We have now shown that the Aβ‐dependent upregulation of the ECE2‐ET‐1 axis in AD occurs at an early stage in the disease and contributes to chronic hypoperfusion of the cerebral cortex, and to a lesser extent the underlying WM. Our findings raise the possibility of benefit from EDN1 receptor antagonists in reducing the deleterious effects of long‐term cerebral hypoperfusion mediated by the activation of EDN1A receptors on vascular smooth muscle cells. Bosentan, a dual EDN1A/B receptor antagonist, preserved aortic and carotid endothelial function in Tg2576 mice, which overexpress APP 15, and EDN1A receptor antagonists have been shown to have therapeutic benefit in several peripheral diseases associated with abnormal activation of the endothelin system: pulmonary hypertension, some forms of renal disease, systemic arterial hypertension, heart failure, allograft rejection and diabetes/insulin resistance (reviewed in 44). Whilst EDN1A receptor antagonists would not address the presumed underlying cause of AD, the present data suggest that these drugs have the potential to ameliorate some of the damaging neurobiological manifestations of the disease from an early stage.

## Supporting information

Additional Supporting Information may be found in the online version of this article at the publisher's web‐site:

**Figure S1.** (**A**) Bar chart showing elevated VEGF in relation to disease severity in the precuneus. Control and AD cases were grouped according to Braak tangle stage, irrespective of dementia status. *Post hoc* analysis showed that VEGF level was significantly higher in the V–VI than the 0–II group. Scatterplot showing positive correlation between VEGF and insoluble Aβ42 (*r* = 0.41) (**B**) but not Aβ40 (**C**) in the precuneus. (**D**) Bar chart showing lower ACE activity in early AD (Braak stage III–IV) than in late stage disease (Braak stage V–VI). **P < 0.01.

**Tables S1 and S2.** The demographic data, neuropathological findings, and MRC identifier numbers in this cohort are summarized.
