# Supplementary figures and images for: Pathophysiology of Hypoperfusion of the Precuneus in Early Alzheimer's Disease
Source: Brain Pathol. 2015 Nov 9;26(4):533–41. doi: 10.1111/bpa.12331 (PMC4982069; doi:10.1111/bpa.12331)

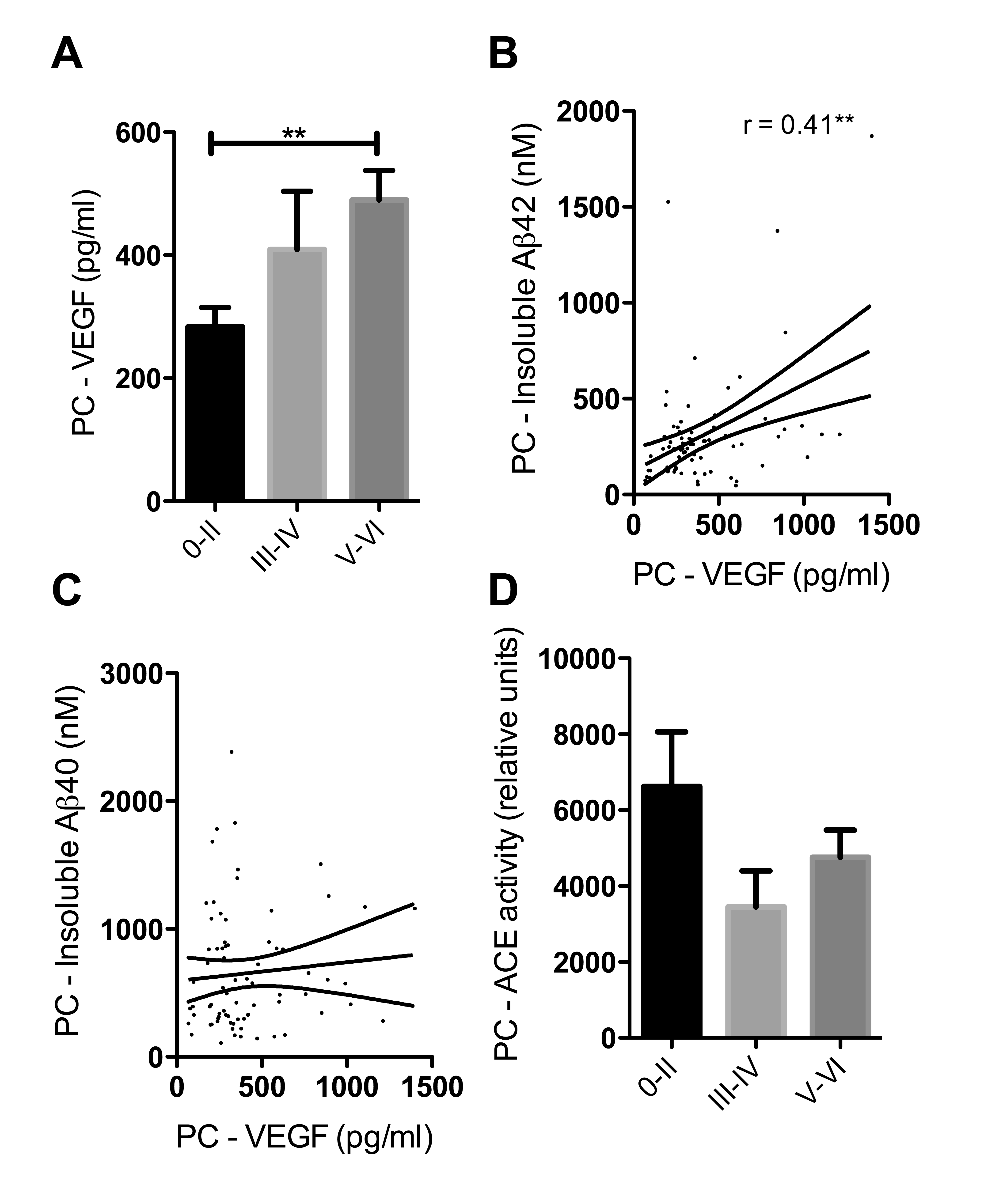

Supplement: Supplementary file 1 — Figure S1. (A) Bar chart showing elevated VEGF in relation to disease severity in the precuneus. Control and AD cases were grouped according to Braak tangle stage, irrespective of dementia status. Post hoc analysis showed that VEGF level was significantly higher in the V–VI than the 0–II group. Scatterplot showing positive correlation between VEGF and insoluble Aβ42 (r = 0.41) (B) but not Aβ40 (C) in the precuneus. (D) Bar chart showing lower ACE activity in early AD (Braak stage III–IV) than in late stage disease (Braak stage V–VI). **P < 0.01. [file BPA-26-533-s001.tiff]
