# Supplementary material for: Pathophysiology of Hypoperfusion of the Precuneus in Early Alzheimer's Disease
Source: Brain Pathol. 2015 Nov 9;26(4):533–41. doi: 10.1111/bpa.12331 (PMC4982069; doi:10.1111/bpa.12331)
Supplement: Supplementary file 2 — Tables S1 and S2. The demographic data, neuropathological findings, and MRC identifier numbers in this cohort are summarized. [file BPA-26-533-s002.docx]

**Supplementary table 1**

|  |  | **AD** (n = 70) | **Control** (n = 37) |
| --- | --- | --- | --- |
| **Age (y ± SD)** |  | 79.8 **±** 8.3 | 79.8 **±** 8.7 |
| **Gender (F:M)** |  | 42:28 | 12:25 |
| **PM delay (h ± SD)** |  | 36.7 **±** 21.5 | 35.0 **±** 15.4 |
| **Braak tangle stage** | 0 | 0 | 4 |
|  | I | 0 | 7 |
|  | II | 0 | 21 |
|  | III | 0 | 5 |
|  | IV | 8 | 0 |
|  | V | 29 | 0 |
|  | VI | 33 | 0 |
| **SVD score^#^** | 0 | 7 | 7 |
|  | 1 | 25 | 21 |
|  | 2 | 26 | 8 |
|  | 3 | 7 | 1 |
| **CAA score^*^** | 0 | 19 | 28 |
|  | 1 | 15 | 4 |
|  | 2 | 13 | 5 |
|  | 3 | 18 | 0 |

^#^ 5 cases in AD group with unavailable SVD scores

**^*^** 5 cases in AD group with unavailable CAA scores

**Supplementary table 2**

| **Diagnosis** | **Age**  **at death**  **(y)** | **Gender** | **Post-mortem**  **delay (h)** | **Braak tangle stage** | **Small**  **vessel**  **disease**  **score** | **Cerebral**  **amyloid**  **angiopathy**  **score** | **MRC identifier** |
| --- | --- | --- | --- | --- | --- | --- | --- |
| Control | 78 | F | 24 | II | 2 | 1 | BBN_8671 |
| Control | 64 | M | 12 | II | 0 | 0 | BBN_8700 |
| Control | 58 | M | 20 | 0 | 0 | 0 | BBN_8702 |
| Control | 72 | M | 42 | I | 1 | 0 | BBN_8706 |
| Control | 90 | M | 45 | II | 0 | 0 | BBN_8708 |
| Control | 77 | M | 55 | I | 1 | 0 | BBN_8717 |
| Control | 78 | M | 12 | II | 1 | 0 | BBN_8722 |
| Control | 80 | M | 67 | III | 2 | 2 | BBN_8723 |
| Control | 73 | M | 36 | II | 0 | 0 | BBN_8725 |
| Control | 93 | F | 18 | II | 1 | 0 | BBN_8739 |
| Control | 82 | M | 30 | II | 2 | 0 | BBN_8751 |
| Control | 75 | M | 48 | II | 1 | 0 | BBN_8759 |
| Control | 73 | M | 33 | I | 1 | 0 | BBN_8776 |
| Control | 69 | M | 66 | II | 1 | 0 | BBN_8779 |
| Control | 73 | F | 59 | I | 1 | 0 | BBN_8835 |
| Control | 90 | M | 40 | III | 1 | 0 | BBN_8883 |
| Control | 83 | F | 24 | II | 0 | 0 | BBN_8898 |
| Control | 82 | M | 3 | II | 1 | 0 | BBN_8923 |
| Control | 72 | F | 24 | 0 | 2 | 0 | BBN_8980 |
| Control | 78 | M | 48 | I | 1 | 0 | BBN_8983 |
| Control | 76 | M | 23 | II | 1 | 0 | BBN_9028 |
| Control | 73 | M | 35 | III | 1 | 0 | BBN_9292 |
| Control | 90 | M | 5.5 | II | 1 | 0 | BBN_9299 |
| Control | 93 | M | 37.75 | III | 2 | 1 | BBN_9311 |
| Control | 80 | M | 45.75 | 0 | 2 | 2 | BBN_9329 |
| Control | 94 | F | 21 | II | 2 | 2 | BBN_9340 |
| Control | 92 | M | 34.25 | II | 2 | 0 | BBN_9344 |
| Control | 87 | M | 24 | II | 1 | 1 | BBN_4205 |
| Control | 85 | M | 30.5 | II | 0 | 0 | BBN_9354 |
| Control | 77 | M | 42 | I | 1 | 2 | BBN_9359 |
| Control | 87 | F | 47 | III | 1 | 0 | BBN_4229 |
| Control | 86 | F | 32 | II | 1 | 3 | BBN_9365 |
| Control | 68 | F | 38.75 | 0 | 0 | 0 | BBN_9389 |
| Control | 73 | F | 50 | II | 1 | 0 | BBN_9399 |
| Control | 90 | F | 41 | II | 1 | 2 | BBN_9407 |
| Control | 87 | M | 42 | II | 3 | 0 | BBN_9408 |
| Control | 74 | F | 39.5 | I | 1 | 1 | BBN_9422 |
| AD | 78 | F | 9 | V | 2 | 0 | BBN_8834 |
| AD | 77 | F | 43 | IV | 0 | 0 | BBN_8848 |
| AD | 71 | M | 30 | VI | 2 | 2 | BBN_8910 |
| AD | 82 | F | 24 | VI | 1 | 3 | BBN_8912 |
| AD | 75 | F | 40 | VI | 1 | 3 | BBN_8921 |
| AD | 74 | F | 12 | VI | 1 | 3 | BBN_8997 |
| AD | 89 | F | 4 | VI | 1 | 3 | BBN_9005 |
| AD | 79 | M | 28 | VI | 1 | 1 | BBN_9026 |
| AD | 65 | M | 27 | VI | 1 | 2 | BBN_9030 |
| AD | 85 | M | 66 | VI | 1 | 3 | BBN_9031 |
| AD | 86 | M | 48 | VI | 2 | 0 | BBN_9044 |
| AD | 57 | F | 24 | V | 1 | 0 | BBN_9052 |
| AD | 84 | F | 20 | V | 3 | 0 | BBN_9076 |
| AD | 93 | M | 20 | VI | 1 | 0 | BBN_9106 |
| AD | 74 | F | 52.5 | V | 1 | 3 | BBN_9112 |
| AD | 83 | F | 5 | V | 2 | 0 | BBN_9122 |
| AD | 74 | F | 35 | V | 2 | 3 | BBN_9123 |
| AD | 77 | F | 26 | VI | 1 | 3 | BBN_9136 |
| AD | 79 | M | 27 | VI | 2 | 1 | BBN_9155 |
| AD | 63 | M | 43 | VI | 0 | 0 | BBN_9162 |
| AD | 69 | F | 71 | VI | 1 | 3 | BBN_9163 |
| AD | 92 | F | 24 | V | 0 | 3 | BBN_9164 |
| AD | 86 | F | 31 | V | 2 | 2 | BBN_9173 |
| AD | 64 | M | 9 | VI | 1 | 1 | BBN_9179 |
| AD | 80 | F | 48 | V | 1 | 2 | BBN_9181 |
| AD | 74 | M | 24 | V | 2 | 1 | BBN_9182 |
| AD | 75 | F | 21 | VI | 1 | 1 | BBN_9186 |
| AD | 78 | F | 21 | VI | 2 | 3 | BBN_9189 |
| AD | 89 | F | 39 | V | 2 | 3 | BBN_9194 |
| AD | 77 | F | 14 | VI | 2 | 0 | BBN_9198 |
| AD | 84 | M | 64 | V |  |  | BBN_9200 |
| AD | 85 | F | 85 | VI | 2 | 3 | BBN_9205 |
| AD | 88 | F | 75 | V |  |  | BBN_9243 |
| AD | 83 | F | 85 | VI |  |  | BBN_9248 |
| AD | 82 | F | 110 | IV |  |  | BBN_9257 |
| AD | 83 | M | 48 | V | 2 | 2 | BBN_9261 |
| AD | 81 | M | 4 | VI | 2 | 3 | BBN_9262 |
| AD | 74 | M | 48 | V | 3 | 1 | BBN_9263 |
| AD | 80 | M | 72 | V | 1 | 2 | BBN_9266 |
| AD | 78 | M | 49 | VI | 0 | 2 | BBN_9274 |
| AD | 87 | M | 36 | VI | 1 | 0 | BBN_9275 |
| AD | 76 | M | 11 | V | 3 | 0 | BBN_9280 |
| AD | 82 | F |  | V |  |  | BBN_9283 |
| AD | 85 | M | 49.5 | VI | 3 | 0 | BBN_9295 |
| AD | 69 | M | 12 | V | 1 | 0 | BBN_9303 |
| AD | 89 | F | 27.5 | V | 2 | 1 | BBN_9308 |
| AD | 67 | F | 24.25 | VI | 2 | 1 | BBN_9315 |
| AD | 84 | F | 20.5 | VI | 3 | 2 | BBN_9323 |
| AD | 80 | F | 50.75 | V | 2 | 2 | BBN_9341 |
| AD | 65 | F | 11.5 | VI | 1 | 2 | BBN_9342 |
| AD | 80 | M | 24 | IV | 1 | 1 | BBN_9343 |
| AD | 88 | F | 64 | VI | 1 | 1 | BBN_9346 |
| AD | 64 | M | 66.5 | V | 0 | 0 | BBN_4202 |
| AD | 65 | M | 38.5 | V | 2 | 0 | BBN_4204 |
| AD | 80 | F | 26 | IV | 0 | 2 | BBN_4215 |
| AD | 84 | F | 48 | VI | 2 | 0 | BBN_4216 |
| AD | 83 | M | 11 | V | 1 | 1 | BBN_9361 |
| AD | 77 | M | 19 | VI | 1 | 3 | BBN_9367 |
| AD | 73 | F | 50.5 | V | 1 | 1 | BBN_9371 |
| AD | 84 | F | 22 | V | 2 | 1 | BBN_9378 |
| AD | 82 | F | 39.5 | VI | 2 | 1 | BBN_9379 |
| AD | 81 | M | 32 | IV | 3 | 1 | BBN_9394 |
| AD | 97 | F | 36 | IV | 2 | 0 | BBN_9397 |
| AD | 86 | F | 45.25 | VI | 2 | 3 | BBN_9401 |
| AD | 78 | F | 61 | VI | 1 | 0 | BBN_9417 |
| AD | 88 | F | 26 | VI | 3 | 2 | BBN_9419 |
| AD | 77 | M | 21.75 | VI | 0 | 2 | BBN_9421 |
| AD | 88 | M | 36 | IV | 2 | 3 | BBN_9433 |
| AD | 90 | F | 46.75 | V | 1 | 3 | BBN_14398 |
| AD | 99 | F | 43 | V | 2 | 0 | BBN_14404 |
| AD | 88 | F | 42.5 | V | 2 | 1 | BBN_19614 |
